# Increase in Reported Cholera Cases in Haiti Following Hurricane Matthew: An Interrupted Time Series Model

**DOI:** 10.4269/ajtmh.17-0964

**Published:** 2018-12-26

**Authors:** Erin Hulland, Saleena Subaiya, Katilla Pierre, Nickolson Barthelemy, Jean Samuel Pierre, Amber Dismer, Stanley Juin, David Fitter, Joan Brunkard

**Affiliations:** 1 Division of Global Health Protection, Center for Global Health, Centers for Disease Control and Prevention, Atlanta, Georgia;; 2 Global Immunization Division, Center for Global Health, Centers for Disease Control and Prevention, Atlanta, Georgia;; 3 MSPP Haiti, Directorate of Epidemiology, Laboratory and Research, Delmas, Haiti;; 4 Centers for Disease Control and Prevention Haiti, Tabarre, Haiti;; 5 Division of Foodborne, Waterborne and Environmental Diseases, National Center for Emerging and Zoonotic Infectious Diseases, Centers for Disease Control and Prevention, Atlanta, Georgia

## Abstract

Matthew, a category 4 hurricane, struck Haiti on October 4, 2016, causing widespread flooding and damage to buildings and crops, and resulted in many deaths. The damage caused by Matthew raised concerns of increased cholera transmission particularly in Sud and Grand’Anse departments, regions which were hit most heavily by the storm. To evaluate the change in reported cholera cases following Hurricane Matthew on reported cholera cases, we used interrupted time series regression models of daily reported cholera cases, controlling for the impact of both rainfall, following a 4-week lag, and seasonality, from 2013 through 2016. Our results indicate a significant increase in reported cholera cases after Matthew, suggesting that the storm resulted in an immediate surge in suspect cases, and a decline in reported cholera cases in the 46-day post-storm period, after controlling for rainfall and seasonality. Regression models stratified by the department indicate that the impact of the hurricane was regional, with larger surges in the two most highly storm-affected departments: Sud and Grand’Anse. These models were able to provide input to the Ministry of Health in Haiti on the national and regional impact of Hurricane Matthew and, with further development, could provide the flexibility of use in other emergency situations. This article highlights the need for continued cholera prevention and control efforts, particularly in the wake of natural disasters such as hurricanes, and the continued need for intensive cholera surveillance nationally.

## INTRODUCTION

In October 2010, 10 months after the devastating earthquake in metropolitan Port-au-Prince in January, the first cholera cases, later confirmed as *Vibrio cholera* O1 (serotype Ogawa, biotype El Tor), were reported in Haiti in the Artibonite and Centre administrative departments.^1^ In the next 2 years, > 600,000 cholera cases (suspect and laboratory-confirmed) were reported through the Ministry of Public Health and Population’s (French Acronym MSPP) National Cholera Surveillance System (NCSS).^1–3^ In 2016, 41,421 cholera cases were reported to NCSS.^3^ Although the MSPP’s Directorate of Epidemiology, Laboratory, and Research, which is responsible for NCSS, has reported a reduction in the number of cases in recent years, cholera continues to be reported daily from almost all of Haiti’s 10 departments and transmission has recently been considered endemic in Haiti.^4–6^

Hurricane Matthew, a category 4 hurricane, made landfall in Haiti on October 4, 2016, resulting in extensive flooding, infrastructure damage, crop loss, and many deaths.^2^ Haiti’s Grand’Anse and Sud departments, both located in the southwestern tip of Hispaniola, were the most affected departments, with the closure of health facilities, lack of electricity, and shortages of clean water sources leading to increased concern for a secondary cholera epidemic.

In the weeks following Hurricane Matthew, health facilities in Sud and Grand’Anse began to report an increase in cases of acute watery diarrhea, raising the alarm of possible increased cholera transmission.^7^ To determine whether an increase in cholera suspect cases associated with Hurricane Matthew, we modeled the impact of Hurricane Matthew on reported cholera suspect cases both nationally and by department, controlling for seasonal trends and rainfall.

## METHODS

### Data sources.

In NCSS, a suspect cholera case is defined as a person presenting with acute watery diarrhea, with or without vomiting, to a health facility. We analyzed NCSS data from January 2013 to November 19, 2016.^7^ Because of the higher incidence of diarrheal disease from causes other than cholera in young children, we included only suspect cholera cases among persons aged 5 years and older. In addition, the time period of analysis was restricted to include cases after cholera was considered endemic in Haiti, from January 2013 to November 2016.

Estimated daily rainfall data were acquired from the National Oceanic and Atmospheric Administration Center for Satellite Applications and Research (STAR).^8^ The rainfall data were presented as average millimeters of daily precipitation of all STAR raster data points within a department. Each department, therefore, had daily-averaged rainfall data for use in the models, except where no data were available, which were considered missing.

### Data analysis.

Data from the cholera surveillance system and rainfall database were imported using R software for cleaning and analysis.^9^ R Markdown codes were created within 2 weeks of the hurricane to automate two different processes: the generation of rate ratios from week-to-week and year-to-year at the departmental and the interrupted time series (ITS) regression models at the national and departmental level. The comparison of institution-collected data versus aggregated departmental-level data is not presented in this article; all data presented were obtained from the aggregated departmental-level data from NCSS.^10^

**Figure 1.**
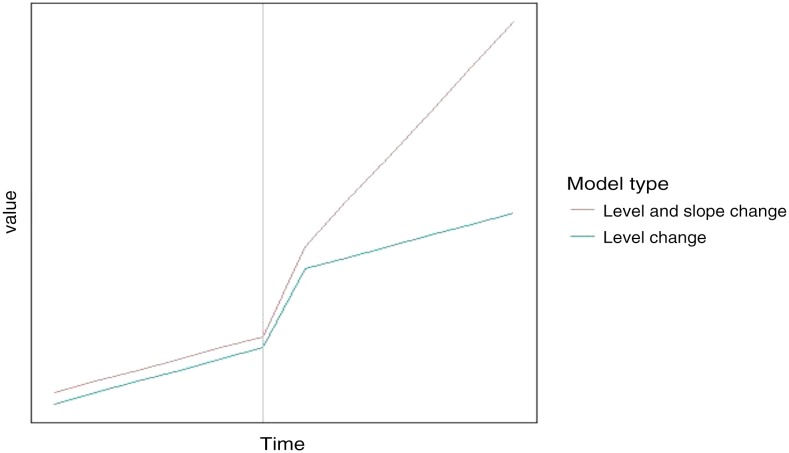
Two types of interrupted time series regression models used for Hurricane Matthew analysis. The data used in this figure are a theoretical representation of the level-and-slope change model (in red) and the level change model (in blue) and do not represent study data. The grey horizontal line is meant to reflect the intervention period, such as the hurricane in our study. This figure appears in color at www.ajtmh.org.

We used ITS regression models using national data to assess the overall impact of Hurricane Matthew on the number of reported cholera cases. Interrupted time series regression models use segmented regression and are used to assess statistical significance of an event with a clearly defined pre- and post-event period. These methods have been used previously to evaluate the impact of public health interventions where randomized controlled trials are not feasible. Interrupted time series regression has also been applied to assess the impact of environmental factors on health outcomes.^11–15^ To compare patterns of cholera cases in regions most heavily affected by Matthew—primarily the Sud and Grand’Anse departments—with those that were not affected, we constructed 10 departmental level models, one for each department in Haiti. Suspect cholera case counts were assumed to follow a quasi-Poisson distribution which allows overdispersion (to overcome the assumption of the Poisson model that variance is equal to the expected case count).^16^ In our models, we considered both a level change—an abrupt rise in cases immediately following the hurricane—and a level-and-slope change—a gradual change in cases over time following the hurricane—while controlling for seasonal variability and rainfall patterns to assess the immediate but also longer term impacts of the hurricane on cholera in Haiti. In Figure 1, the blue line represents a level change alone, whereas the red line considers both a level and slope change, the type of model we used for our ITS regression models.

Each of the 11 generalized linear models (one for a national level, 10 for 10 departments) included time, a continuous numeric variable representing each day since January 1, 2013, average departmental daily rainfall, a dichotomous indicator of the time pre- and post-Hurricane Matthew to assess a level change, and an interaction term between time and the Hurricane Matthew indicator to assess a level-and-slope change. Seasonality was modeled in Fourier terms as a single pair of sine and cosine functions to account for the year-long seasonal pattern seen in Haiti.^15^ Rainfall was modeled using a 4-week lag, a value chosen based on previous research suggesting lags between 0 and 8 weeks, and in particular a 4-week lag in Haiti^17^; rainfall, reported in average millimetre per day per department, was scaled by 0.1 for interpretability.^4,11,17–21^ Equation 1 identifies the model used for the analysis, where µ is the expected daily suspected cholera case counts at time *t*, either nationally or departmentally, dependent on the model; ln (POP) is an offset used to account for the population *t* risk; β_0_ is the intercept, or baseline case counts; Time_*t*_ is the number of days since January 1, 2013; Hurricane Matthew_*t*_ (HM_*t*_) is a dichotomous indicator variable where 0 is any time before Hurricane Matthew and 1 is any time following Hurricane Matthew; Rain_t_ is a continuous variable representing average daily rainfall in millimeters, scaled by 0.1; two seasonality Fourier terms—one sine, one cosine—with a seasonal period of 1 year; Time following HM_*t*_ is a continuous variable tracking days following Hurricane Matthew but is 0 for any period before the hurricane; *e*_*t*_ represents the random error at time *t*. All data management, analysis, and visualization were performed using R Studio version 1.0.136.^9^

$$\text{ln}\left({µ}_{t}\right)=\text{ln}\left(\text{POP}\right)+\beta_{0}{+\beta }_{1}{\text{Time}}_{t}{+\beta }_{2}{\text{HM}}_{t}{+\beta }_{3}{\text{Rain}}_{t}{+\beta }_{4}{\left(\text{Seasonality Fourier sine term, 365 day cycle}\right)}_{t}{+\text{β}}_{5}{\left(\text{Seasonality Fourier cosine term, 365 day cycle}\right)}_{t}+\beta_{6}{\left(\text{Time following HM}\right)}_{t}+e_{t}$$ (1)

## RESULTS

**Figure 2.**
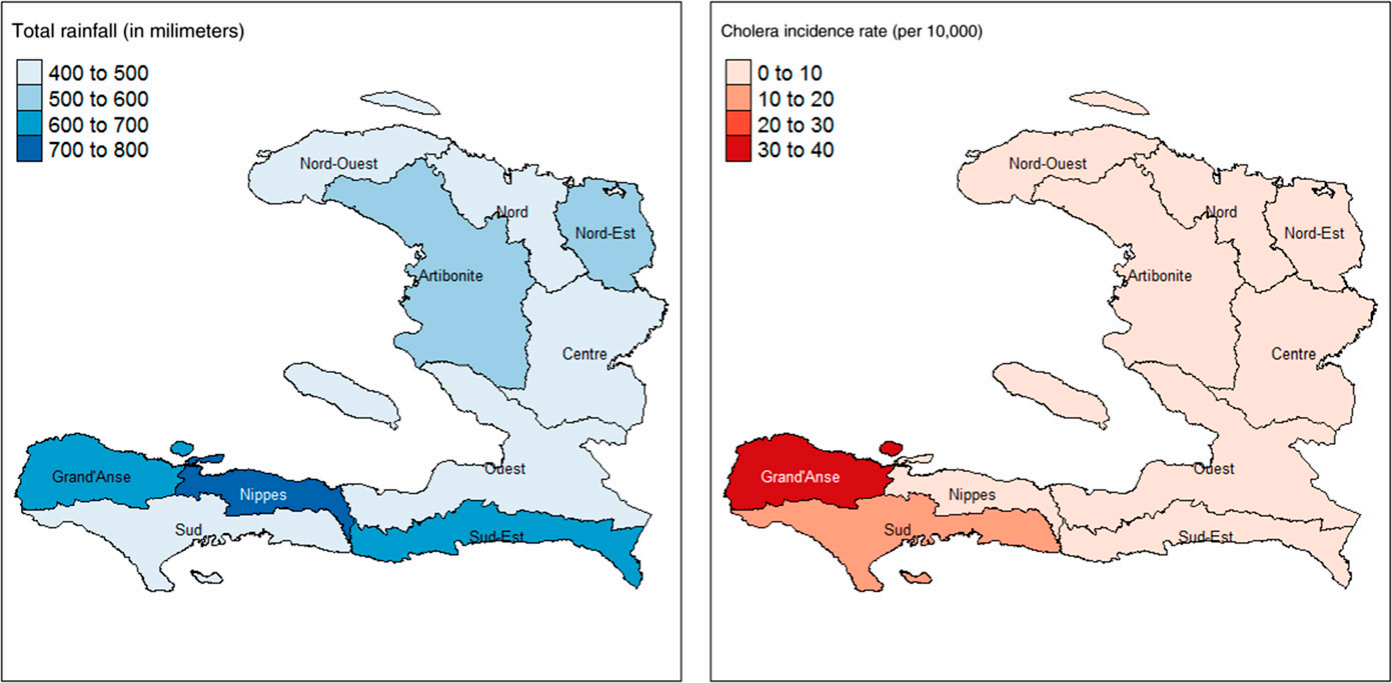
Rainfall and cholera incidence rate in Haiti in the 46-day period during and following Hurricane Matthew. Haiti departmental shapefile obtained from HaitiData.org, published by Centre National de l’Information Geospatiale en Haiti (CNIGS) on May 9, 2012 and accessed on May 9, 2017. Total rainfall expressed as millimetre over the 46-day period per department. Cholera incidence rate expressed as number of cases over 5 years per population per 46-day period per 10,000 population per department. This figure appears in color at www.ajtmh.org.

The impact of rainfall and cholera incidence rate in the 46-day period following Hurricane Matthew can be seen in Figure 2, with the highest levels of rainfall in the Sud-Est, Nippes, and Grand’Anse departments and highest cholera incidence rates occurring in the Sud and Grand’Anse. A total of 133,092 suspect cholera cases in persons aged 5 years and older were reported between January 1, 2013, to November 19, 2016, of which 6,365 (5%) occurred in the 46-day period following Hurricane Matthew. Average daily cholera case counts in the pre- and post-hurricane period can be seen in Table 1, as well as average daily rainfall and average daily cholera mortality.

**Table 1:** National descriptive characteristics of cholera and rainfall pre- and post-hurricane

	Mean	SD	Median	IQR	Min	Max
Daily reported cholera cases ≥ 5 years of age, pre-hurricane	92.3	56.4	80.0	49.0–126.0	8.0	329.0
Daily reported cholera cases ≥ 5 years of age, post-hurricane	138.4	31.5	133.5	117.2–154.8	83.0	214.0
Daily rainfall (in mm)	15.6	75.7	0.0	0.0–2.7	0.0	1,710.5
Daily cholera-related deaths, pre-hurricane	1.1	1.5	1.0	0.0–2.0	0.0	13.0
Daily cholera-related deaths, post-hurricane	2.4	2.7	2.0	1.0–3.0	0.0	16.0

IQR = interquartile range; Max = maximim; Min = minimum; SD = standard deviation.

**Figure 3.**
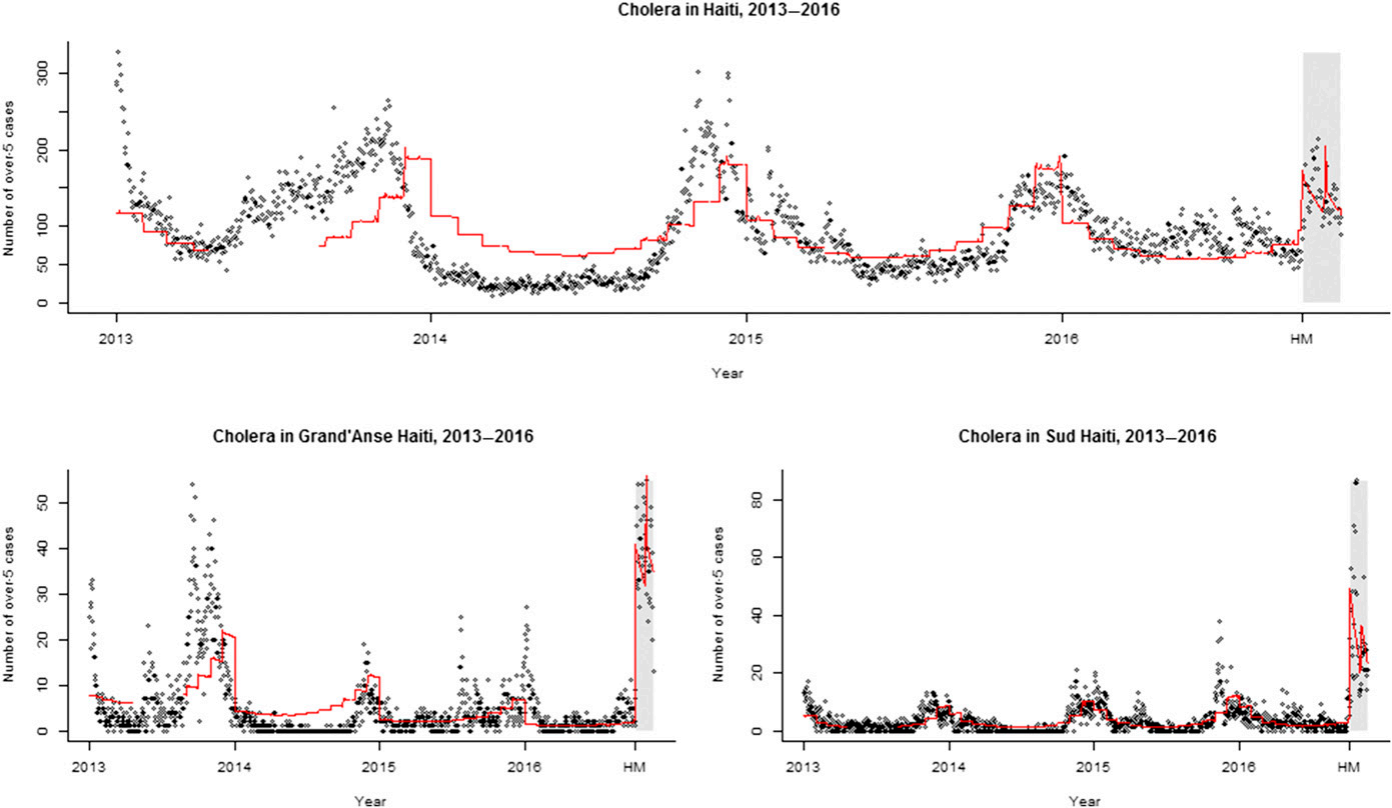
Suspect cholera case counts in Haiti with time series regression models, 2013–2016. HM: Hurricane Matthew; grey box: time since Hurricane Matthew; red line: interrupted time series regression model including both a level change and a slope-and-level change. The top panel presents the national time series cholera data between 2013 and November 2016 with the fitted model in red; the bottom left panel presents the time series cholera data for the Grand’Anse department with the fitted model in red, and the bottom right panel presents the time series cholera data for Sud department with the fitted model in red. This figure appears in color at www.ajtmh.org.

The ITS regression models revealed a significant increase in suspected cholera cases in the period following Hurricane Matthew both nationally (β_2_: 19.6 [95% confidence interval: 7.3–32.0]) and in the two most heavily hurricane-affected departments (Grand’Anse: 14.8 [1.1–28.5], Sud: 41.5 [31.5–51.6]) when compared with the pre-hurricane period, after controlling for rainfall, time, and seasonality, as assessed by the dichotomous Hurricane Matthew indicator (a level change) (Table 1). These models, superimposed over the reported cholera case counts between 2013 and 2016, can be seen in Figure 3, with the period post-hurricane highlighted by the grey box to the far right. The observable significant increase was not uniform across all 10 departments: five of the remaining eight departments did not experience a significant change in case count after Hurricane Matthew, one department experienced a significant decrease in case counts, and two other departments also experienced a significant increase in case counts.

In the national model and in the models for three of the four departments with significant increases post-hurricane—Sud, Centre, and Nord-Ouest—there was a significant downward slope in suspect cholera cases following the initial level change (Table 1), as assessed by the interaction term between time and the dichotomous Hurricane Matthew indicator (the level-and-slope, or post-hurricane slope change). In Grand’Anse, there was no significant observable trend in the post-hurricane slope. The six departments that did not have significant level changes following the hurricane also did not exhibit any post-hurricane slope changes (Table 2).

**Table 2:** Estimated effects of Hurricane Matthew, assessed both as a level change, and as a level-and-slope change

	Level-only change (β_2_)			Level + slope change (β_6_)		
	Estimate	95% CI	*P*-value	Estimate	95% CI	*P*-value
National	**19.623**	**7.298–31.988**	**0.002**	**−0.014**	**−0.023 to −0.005**	**0.002**
Grand’Anse	**14.829**	**1.126–28.521**	**0.034**	**−**0.009	**−**0.018–0.001	0.083
Sud	**141.510**	**31.526–51.553**	**< 0.001**	**−0.028**	**−0.036 to −0.021**	**< 0.001**
Artibonite	13.981	−3.060–31.087	0.108	**−**0.010	**−**0.022–0.003	0.121
Centre	**45.635**	**17.670–74.564**	**0.002**	**−0.033**	**−0.054 to −0.013**	**0.002**
Nord	−9.582	−31.278–11.596	0.380	0.007	**−**0.008–0.022	0.394
Nord-Est	−37.078	−100.409–20.352	0.223	0.026	**−**0.015–0.071	0.228
Nord-Ouest	**40.783**	**7.156–75.027**	**0.018**	**−0.030**	**−0.054 to −0.006**	**0.016**
Nippes	−**66.017**	**−121.227 to −17.756**	**0.012**	**0.047**	**0.013–0.086**	**0.012**
Ouest	23.028	−1.916–48.106	0.071	**−**0.017	**−**0.035–0.001	0.063
Sud-Est	34.438	−5.217–74.792	0.089	**−**0.025	**−**0.054–0.003	0.084

Bold face font indicates statistical significance at the *P* < 0.05 level.

In all of our models, seasonality was significantly related to cholera case counts. Nationally, there was a significant decrease in suspected case counts over the duration of the study period (as measured by the β_1_ coefficient), although this relationship was not uniform across the departments: in those departments that historically were the most impacted by the first cholera epidemic, including Centre and Artibonite, there was a significant downward trend in cases over time; for five other departments, there was a significant upward trend in cases over time. Increased rainfall was significantly related to elevated suspect cholera case counts in two of the 10 departments, Centre and Nord-Est, neither of which were the two primarily impacted departments (See Supplemental File 1).

## DISCUSSION

These models demonstrate that Hurricane Matthew had a significant, immediate impact on reported suspect cholera cases among persons aged 5 years and older; suspect cholera cases increased both at the national level and in the most highly affected departments. These models also suggest that in most of the affected departments, and on a national scale, the suspect cholera cases decreased significantly over the 46-day period following Hurricane Matthew after the observed immediate significant increase, demonstrated by the negative estimates for the level + slope change interaction term. Although not exclusive to Sud and Grand’Anse, the finding that the significant increases in suspected cholera case counts following Hurricane Matthew were not uniform in all departments provides evidence that Hurricane Matthew did have an impact on those highly affected departments or those that have a longstanding history of cholera treatment and transmission, including Centre, a department known for having an established referral hospital and experience with cholera treatment, notable increases in case counts during the rainy season, and high attack rates compared with the rest of the country.^22,23^ Possible reasons for the significant decreasing slopes following the immediate increase in case counts include general seasonal trends and decreased rainfall, heightened reporting in the immediate post-storm period that gradually leveled off, or quick response to the emergency preventing further cases and treating those identified.

We chose to use ITS regression models for this analysis because of their ease of interpretation, rapid code and model development, and the abrupt, clearly defined nature of the hurricane, which allowed us to clearly separate the data into pre- and post-hurricane periods, and because ITS models are most easily implemented with outcomes that change immediately following an event or with a clearly defined lag period, which is true for cholera transmission. We found through this analysis that interrupted time series models are relatively easy to develop and use and allow public health and emergency response personnel to assess in real time how exogenous factors such as hurricanes or other disasters are impacting disease outcomes. As part of the response to Hurricane Matthew, codes were developed in R markdown for future use by Ministry of Health employees, and several trainings in R were conducted so that the models can be used prospectively for other seasonally varying diseases. Furthermore, analyses produced by these models provided evidence of the effect of Hurricane Matthew for the Ministry of Health in Haiti and other emergency response partners. These models highlight the utility of modeling in post-emergency settings and provide a tool for assessing the significance of natural or man-made disasters on disease surveillance.

There are a number of limitations to these models. It is possible that because of heightened awareness of the risk of cholera following the Hurricane, facilities may have incorrectly been reporting other diarrheal cases as suspect cholera cases. To minimize this, we limited our analyses to cases in children and adults aged 5 years and older. In addition, an oral cholera vaccination campaign began in select communes of the Sud and Grand’Anse departments on November 8; case counts may have declined in these departments as a result of vaccination activities, which are not controlled for in our models. Third, hurricane response activities included bolstered local surveillance in the heavily impacted departments (Sud and Grand’Anse) and, thus, reporting of suspect cholera cases may have been intensified in these departments relative to others. Fourth, only seasonality and rain were included as time-varying covariates, but other factors, including migration and temperature, may have impacted the models in ways we were unable to quantify. However, whereas factors such as migration and temperature which are both temporally and theoretically linked to the hurricane have the potential to bias our results, other factors not linked to the hurricane may not have the same impact on the estimates. Fifth, we used one set of Fourier terms to describe seasonality of cholera in our models, but sensitivity analyses carried out at the national level demonstrated that two or more pairs of terms fit the model significantly better. However, each additional pair increased the model fit significantly, but also reduced interpretability of the model, and thus for parsimony, we ultimately chose the model with one singular pair of Fourier terms. Results did not differ substantially when considering multiple pairs of Fourier terms when compared with a single pair. Similarly, Fourier terms are also not able to capture seasonal variability from 1 year to the next. However, although our data reflect slightly variable seasonal patterns annually, we note similarities in the annual pattern with a peak in winter months and a dip in summer months. Finally, the rainfall data were both experimental in their release and missing some data for 2013 and, thus, may not fully represent the true rainfall observed in Haiti over our study evaluation time period.

## CONCLUSION

The national ITS regression model developed in this study suggests an immediate increase in reported cholera cases following the hurricane followed by a significant decline in the post-hurricane period; departmental models further demonstrated that this trend was not uniform for the country and that those more highly impacted departments had significantly higher surges in reported cases following the hurricane. These findings demonstrate that there is a need for continued intensive surveillance following a hurricane as increased cholera transmission is likely. Further research could be beneficial to develop similar models for other infectious or vector-borne diseases following a hurricane or modified for other emergencies. These results of this study revealed that ITS regression models can be developed and implemented relatively quickly in the wake of a natural disaster such as a hurricane and should be considered as part of disaster response to identify where disease transmission was significantly increased to help prevent lives lost and reduce the burden of communicable diseases.

## Supplementary Material

Supplemental files
