# Supplementary material for: Increase in Reported Cholera Cases in Haiti Following Hurricane Matthew: An Interrupted Time Series Model
Source: Am J Trop Med Hyg. 2018 Dec 26;100(2):368–73. doi: 10.4269/ajtmh.17-0964 (PMC6367609; doi:10.4269/ajtmh.17-0964)
Supplement: Supplementary file 1 [file tpmd170964.SD1.pdf]

The following are supplemental materials and will be published online only

Interrupted Time Series Regression Models for Hurricane Matthew

18 September 2018

Summary Statistics

```
##       Date               EPIWEEK          Year       CasesOver5
##  Min.   :2013-01-01   Min.   : 1.00   Min.   :2013   Min.   :  8.00
##  1st Qu.:2013-12-21   1st Qu.:13.00   1st Qu.:2013   1st Qu.: 51.00
##  Median :2014-12-11   Median :26.00   Median :2014   Median : 82.00
##  Mean   :2014-12-11   Mean   :25.99   Mean   :2014   Mean   : 93.79
##  3rd Qu.:2015-11-30   3rd Qu.:39.00   3rd Qu.:2015   3rd Qu.:129.00
##  Max.   :2016-11-19   Max.   :53.00   Max.   :2016   Max.   :329.00
##
##     Rainfall             HM              Month              t
##  Min.   :   0.000   Min.   :0.00000   Min.   : 1.000   Min.   :   1.0
##  1st Qu.:   0.000   1st Qu.:0.00000   1st Qu.: 3.000   1st Qu.: 355.5
##  Median :   0.000   Median :0.00000   Median : 6.000   Median : 710.0
##  Mean   :  15.608   Mean   :0.03242   Mean   : 6.369   Mean   : 710.0
##  3rd Qu.:   2.662   3rd Qu.:0.00000   3rd Qu.: 9.000   3rd Qu.:1064.5
##  Max.   :1710.535   Max.   :1.00000   Max.   :12.000   Max.   :1419.0
##  NA's   :150
##      rain10
##  Min.   :  0.0000
##  1st Qu.:  0.0000
##  Median :  0.0000
##  Mean   :  1.5608
##  3rd Qu.:  0.2662
##  Max.   :171.0535
##  NA's   :150
```

```
##    Min. 1st Qu.  Median    Mean 3rd Qu.    Max.
##     8.0    49.0    80.0    92.3   126.0   329.0
```

```
##    Min. 1st Qu.  Median    Mean 3rd Qu.    Max.
##    83.0   117.2   133.5   138.4   154.8   214.0
```

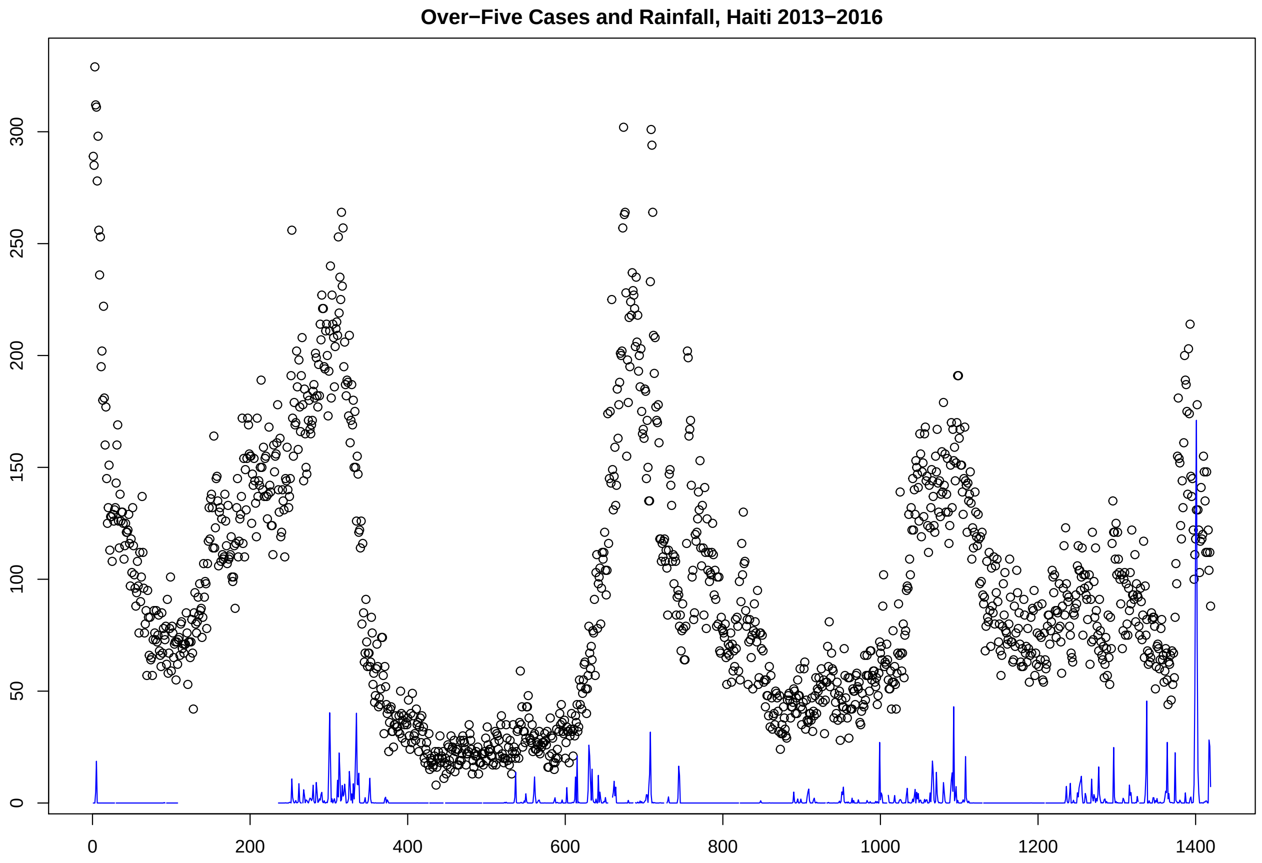

National interrupted time series regression model controlling for rainfall and seasonality, with a level only and slope-and-level change

```
##                                     Estimate       95% LCI
## (Intercept)                     1.736513e+02  1.559676e+02
## t                              -1.032691e-04 -1.811696e-04
## HM                              1.962338e+01  7.297969e+00
## rain10                          1.981405e-03 -1.017957e-03
## harmonic(Month, 1, period = 365)1 -1.805041e+01 -2.009570e+01
## harmonic(Month, 1, period = 365)2 -1.848130e+02 -2.024066e+02
## t:HM                           -1.387243e-02 -2.274059e-02
##                                      95% UCI      P-Value
## (Intercept)                     1.913049e+02 1.071798e-72
## t                              -2.535932e-05 9.480800e-03
## HM                              3.198792e+01 1.863305e-03
## rain10                          4.718260e-03 1.751434e-01
## harmonic(Month, 1, period = 365)1 -1.599964e+01 3.473809e-60
## harmonic(Month, 1, period = 365)2 -1.671902e+02 2.498475e-81
## t:HM                           -5.042507e-03 2.151533e-03
```

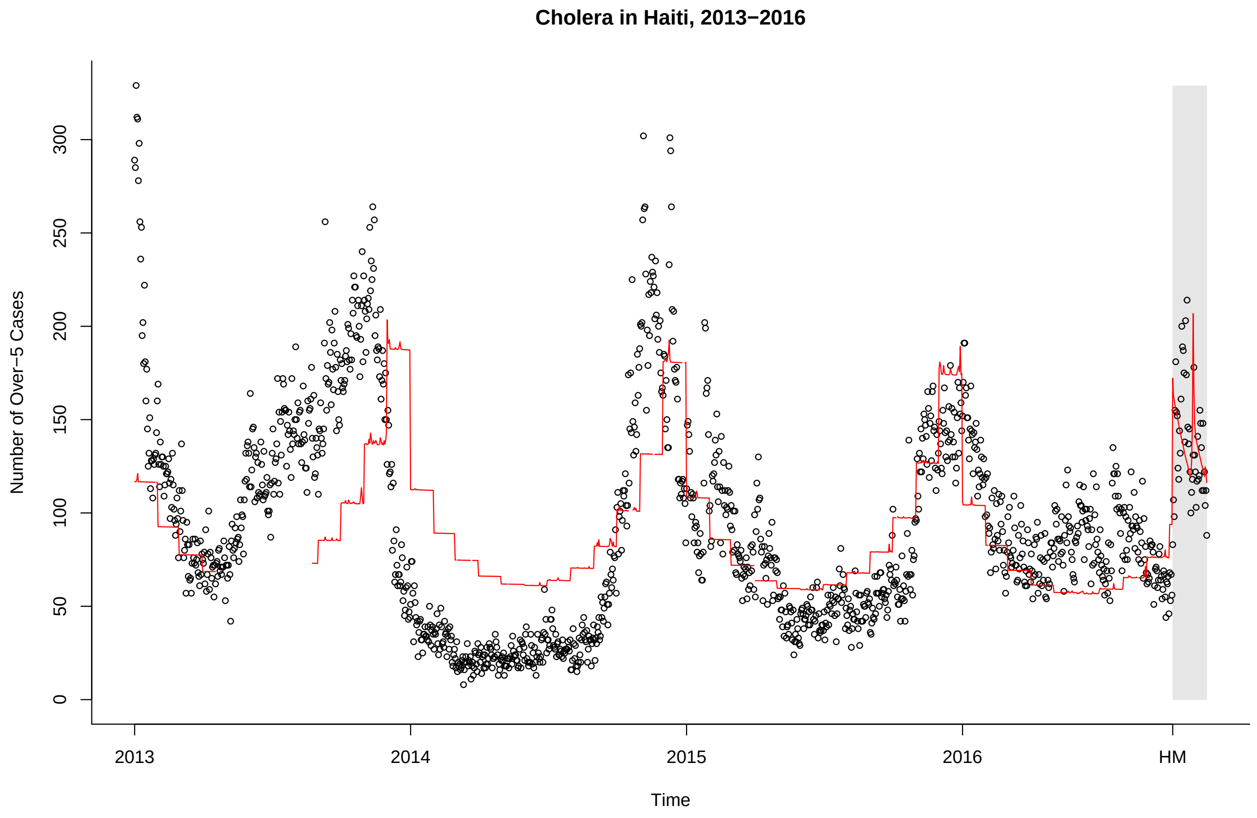

Interrupted time series regression model controlling for rainfall and seasonality, with a level only and slope-and-level change, Grand'Anse

```
##                            Estimate       95% LCI        95% UCI
## (Intercept)              1.320350e+02   86.14089085  177.732727491
## t                       -1.556060e-03   -0.00177247   -0.001342925
## HM                       1.482899e+01    1.12617417   28.521019162
## rain10                   8.226661e-03   -0.01262020    0.025722244
## harmonic(Month, 1, 365)1 -7.839367e+00  -13.32319171   -2.305160458
## harmonic(Month, 1, 365)2 -1.429356e+02 -188.45026482  -97.231837088
## t:HM                    -8.672396e-03   -0.01848964    0.001140641
##                              P-Value
## (Intercept)              1.953859e-08
## t                        1.366606e-42
## HM                       3.388631e-02
## rain10                   3.967788e-01
## harmonic(Month, 1, 365)1 5.351368e-03
## harmonic(Month, 1, 365)2 1.075950e-09
## t:HM                     8.328947e-02
```

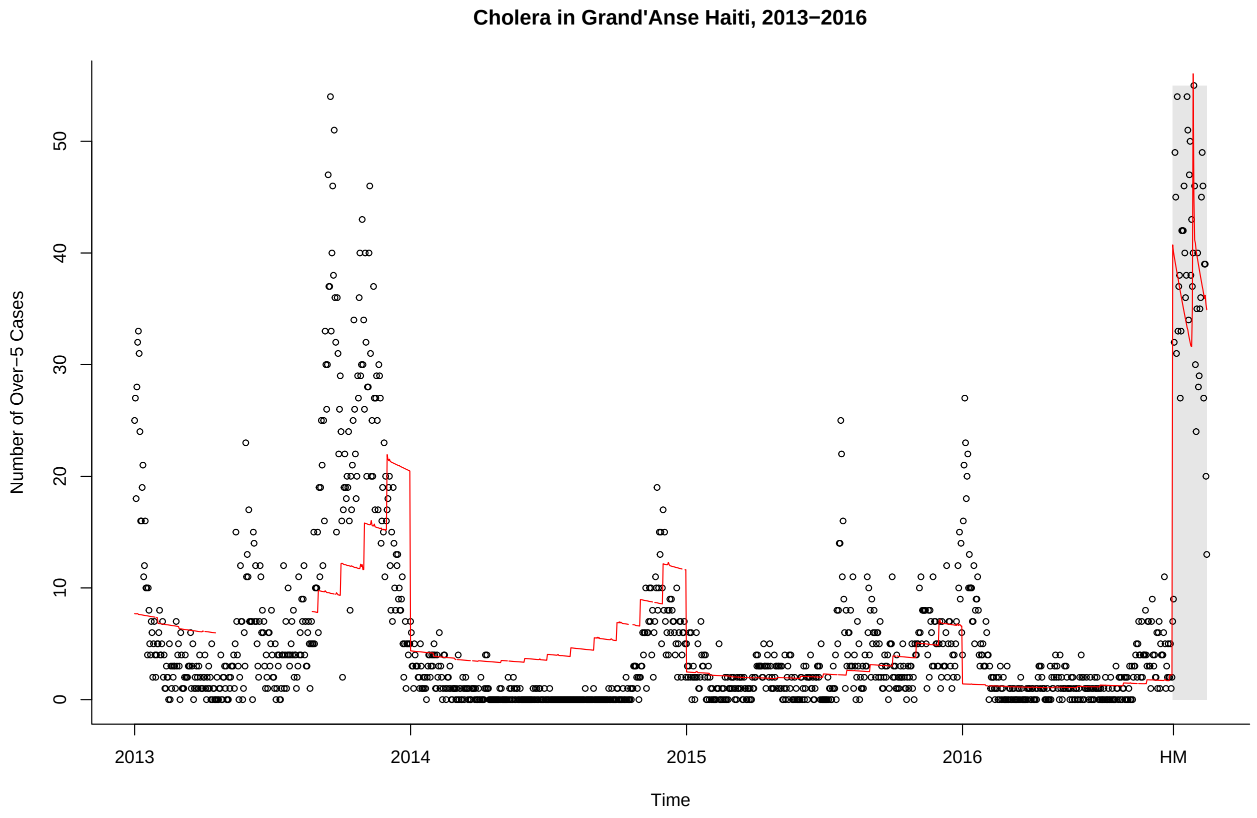

Interrupted time series regression model controlling for rainfall and seasonality, with a level only and slope-and-level change, Sud

```
##                              Estimate       95% LCI       95% UCI
## (Intercept)              4.117314e+02  3.761995e+02  4.474842e+02
## t                        4.758073e-04  3.175301e-04  6.349134e-04
## HM                       4.151047e+01  3.152593e+01  5.155288e+01
## rain10                  -2.399171e-02 -5.940883e-02  8.129131e-03
## harmonic(Month, 1, 365)1 -4.560795e+01 -4.970762e+01 -4.152670e+01
## harmonic(Month, 1, 365)2 -4.229719e+02 -4.586245e+02 -3.875419e+02
## t:HM                    -2.839944e-02 -3.560982e-02 -2.123656e-02
##                               P-Value
## (Intercept)              1.475624e-95
## t                        5.316105e-09
## HM                       1.027262e-15
## rain10                   1.630371e-01
## harmonic(Month, 1, 365)1  4.436773e-90
## harmonic(Month, 1, 365)2 2.209450e-100
## t:HM                     1.898588e-14
```

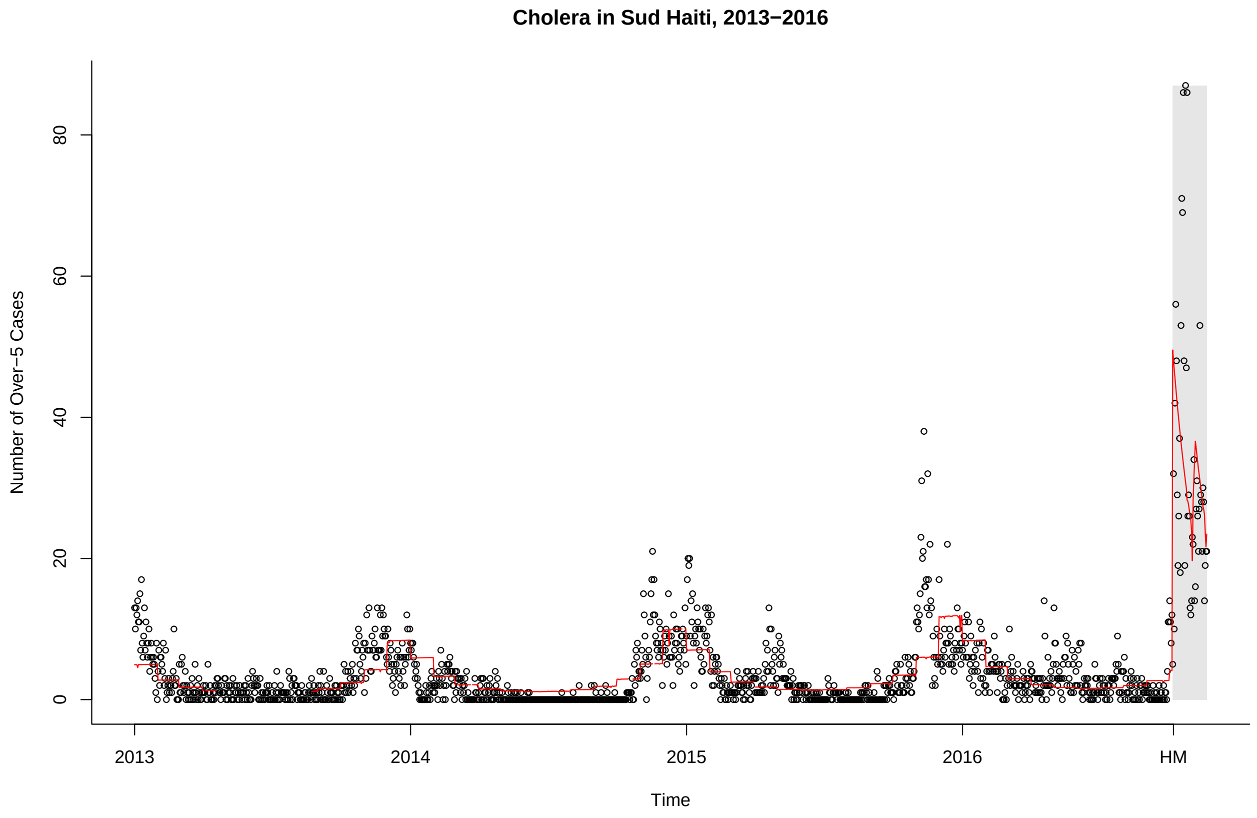

Interrupted time series regression model controlling for rainfall and seasonality, with a level only and slope-and-level change, Artibonite

```
##                                Estimate       95% LCI       95% UCI
## (Intercept)                4.571432e+01  2.331262e+01  6.801238e+01
## t                         -8.836792e-04 -9.814254e-04 -7.863069e-04
## HM                         1.398056e+01 -3.060267e+00  3.108674e+01
## rain10                     1.521619e-02 -2.831065e-02  5.510803e-02
## harmonic(Month, 1, 365)1 -1.990168e+00 -4.606300e+00  6.417069e-01
## harmonic(Month, 1, 365)2 -5.704679e+01 -7.925938e+01 -3.473222e+01
## t:HM                      -9.663862e-03 -2.193533e-02  2.541675e-03
##                                P-Value
## (Intercept)               6.450195e-05
## t                         3.974963e-63
## HM                        1.083061e-01
## rain10                    4.739917e-01
## harmonic(Month, 1, 365)1 1.373609e-01
## harmonic(Month, 1, 365)2 5.834557e-07
## t:HM                      1.214764e-01
```

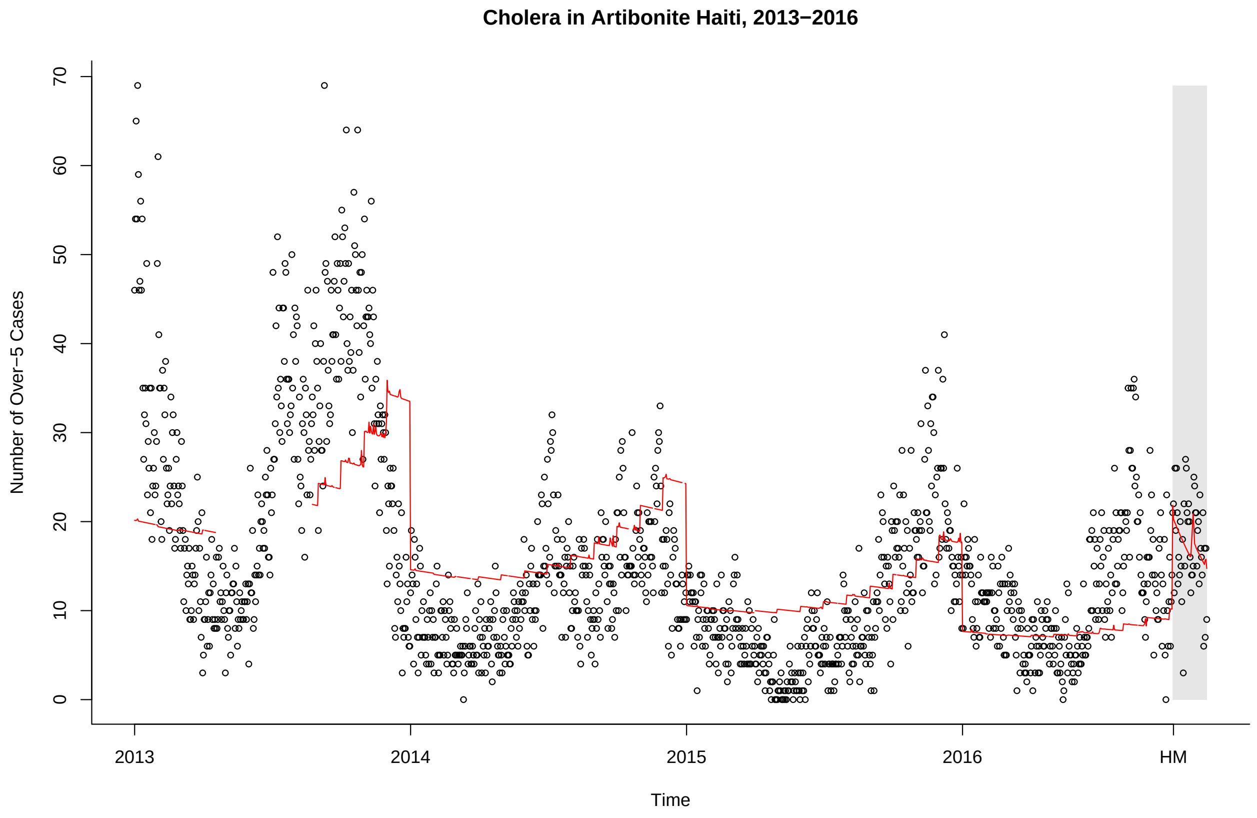

Interrupted time series regression model controlling for rainfall and seasonality, with a level only and slope-and-level change, Centre

```
##                           Estimate        95% LCI       95% UCI
## (Intercept)            -2.551772e+01 -51.917721938  0.6714719910
## t                      -3.575043e-04  -0.000466957 -0.0002481791
## HM                      4.563453e+01  17.670009529 74.5643644076
## rain10                  7.698592e-02   0.030959168  0.1192010227
## harmonic(Month, 1, 365)1  4.166762e+00   1.131034490  7.2301502301
## harmonic(Month, 1, 365)2  1.442572e+01 -11.663641793 40.7234915383
## t:HM                   -3.308573e-02  -0.053907330 -0.0130055109
##                          P-Value
## (Intercept)            5.736779e-02
## t                      2.103008e-10
## HM                     1.646499e-03
## rain10                 6.333316e-04
## harmonic(Month, 1, 365)1 7.496748e-03
## harmonic(Month, 1, 365)2 2.805608e-01
## t:HM                   1.502720e-03
```

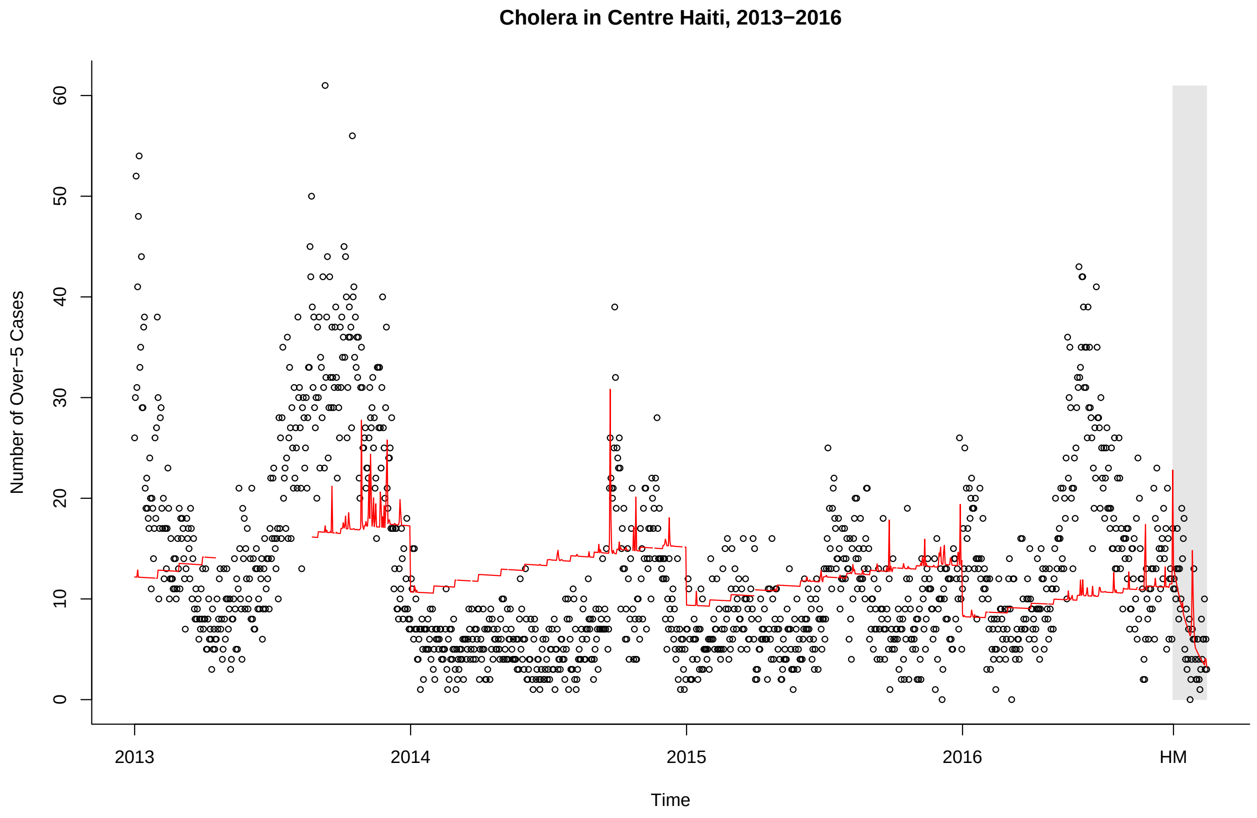

Interrupted time series regression model controlling for rainfall and seasonality, with a level only and slope-and-level change, Nord

```
##                              Estimate       95% LCI       95% UCI
## (Intercept)              2.733884e+02  2.519026e+02  2.948835e+02
## t                       -5.168241e-05 -1.447256e-04  4.139269e-05
## HM                      -9.582108e+00 -3.127758e+01  1.159578e+01
## rain10                   2.926554e-02 -2.331581e-02  7.648691e-02
## harmonic(Month, 1, 365)1 -3.189241e+01 -3.432084e+01 -2.946453e+01
## harmonic(Month, 1, 365)2 -2.836329e+02 -3.050655e+02 -2.622104e+02
## t:HM                     6.650024e-03 -8.496216e-03  2.213696e-02
##                              P-Value
## (Intercept)              6.573015e-112
## t                        2.765469e-01
## HM                       3.801313e-01
## rain10                   2.500360e-01
## harmonic(Month, 1, 365)1 8.023108e-118
## harmonic(Month, 1, 365)2 2.679627e-119
## t:HM                     3.939472e-01
```

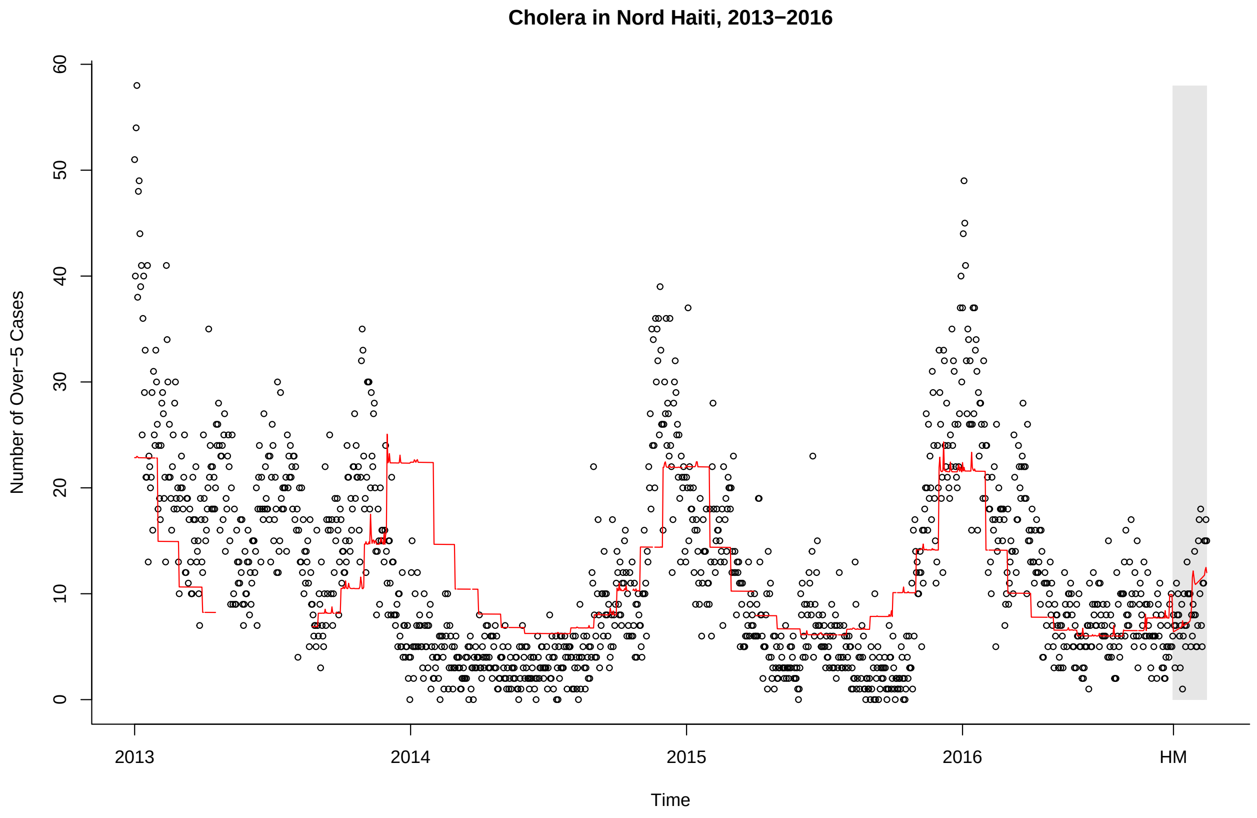

Interrupted time series regression model controlling for rainfall and seasonality, with a level only and slope-and-level change, Nord-Est

```
##                              Estimate       95% LCI       95% UCI
## (Intercept)              1.321450e+02  8.433532e+01  1.796517e+02
## t                       -3.931065e-04 -5.992840e-04 -1.875556e-04
## HM                      -3.707763e+01 -1.004089e+02  2.035207e+01
## rain10                   1.350850e-01  7.106369e-02  1.934332e-01
## harmonic(Month, 1, 365)1 -1.522616e+01 -2.063858e+01 -9.773653e+00
## harmonic(Month, 1, 365)2 -1.436159e+02 -1.909681e+02 -9.596614e+01
## t:HM                     2.611500e-02 -1.494785e-02  7.118444e-02
##                              P-Value
## (Intercept)              6.523326e-08
## t                        1.894216e-04
## HM                       2.225495e-01
## rain10                   1.282783e-05
## harmonic(Month, 1, 365)1 4.715999e-08
## harmonic(Month, 1, 365)2 3.957361e-09
## t:HM                     2.283769e-01
```

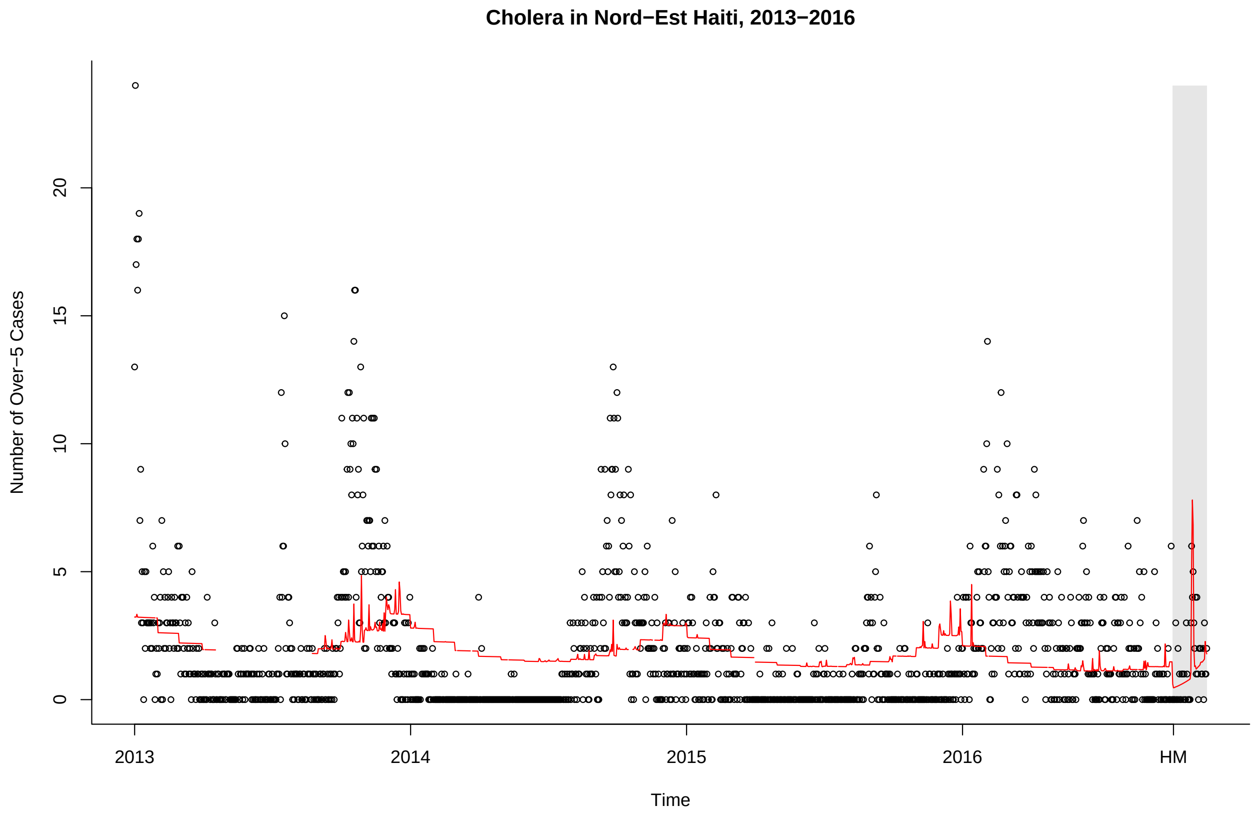

Interrupted time series regression model controlling for rainfall and seasonality, with a level only and slope-and-level change, Nord-Ouest

```
##                              Estimate        95% LCI        95% UCI
## (Intercept)              3.136806e+02  2.796633e+02  3.477597e+02
## t                        4.759768e-04  3.219191e-04  6.308277e-04
## HM                       4.078288e+01  7.156385e+00  7.502697e+01
## rain10                   4.424363e-02 -3.502814e-02  1.139612e-01
## harmonic(Month, 1, 365)1 -3.357267e+01 -3.752192e+01 -2.962125e+01
## harmonic(Month, 1, 365)2 -3.251596e+02 -3.591332e+02 -2.912503e+02
## t:HM                     -2.981247e-02 -5.442070e-02 -5.722327e-03
##                              P-Value
## (Intercept)              4.952071e-65
## t                        2.011162e-09
## HM                       1.803863e-02
## rain10                   2.431753e-01
## harmonic(Month, 1, 365)1 1.737531e-56
## harmonic(Month, 1, 365)2 1.392200e-69
## t:HM                     1.598960e-02
```

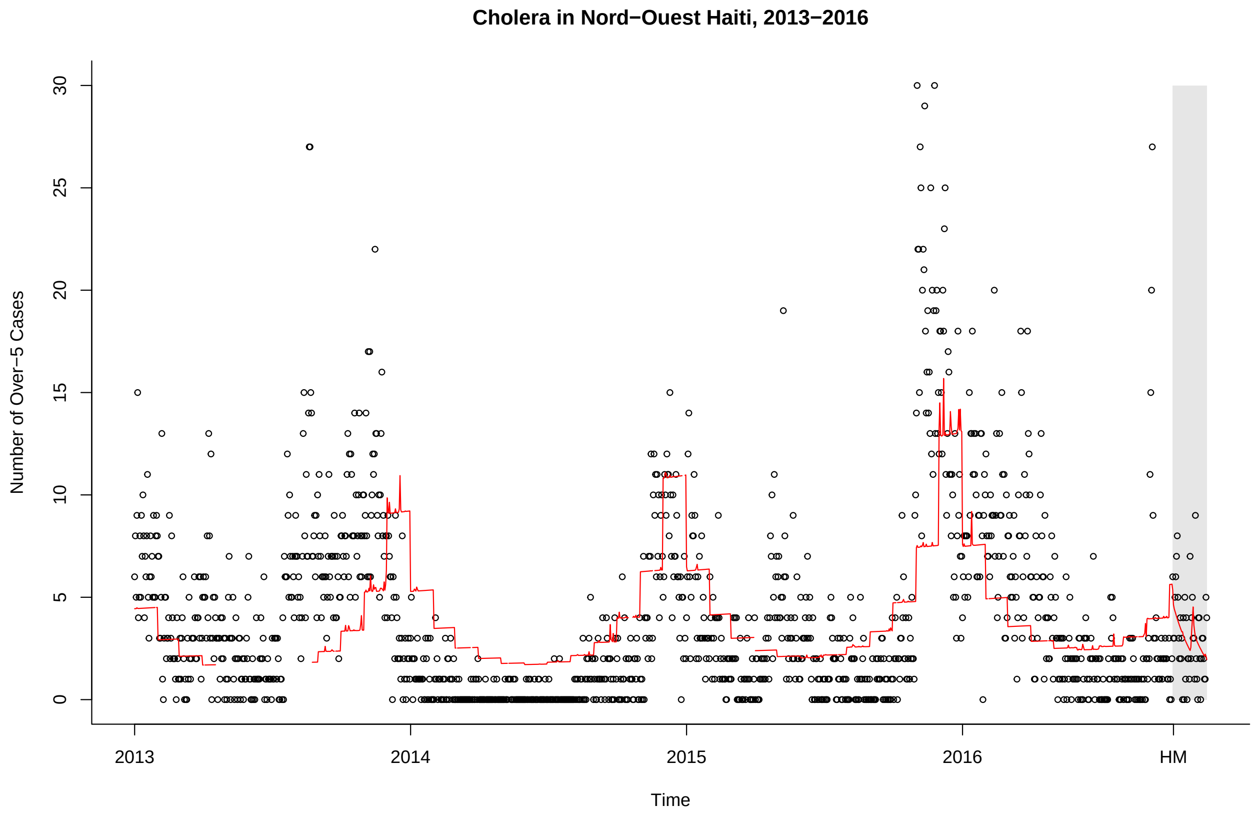

Interrupted time series regression model controlling for rainfall and seasonality, with a level only and slope-and-level change, Nippes

```
##                               Estimate       95% LCI       95% UCI
## (Intercept)                2.548635e+02  1.909670e+02  3.185189e+02
## t                          1.124181e-03  8.261232e-04  1.428462e-03
## HM                        -6.601726e+01 -1.212267e+02 -1.775565e+01
## rain10                    -6.016898e-02 -1.962008e-01  2.621919e-02
## harmonic(Month, 1, 365)1 -3.022139e+01 -3.740345e+01 -2.300567e+01
## harmonic(Month, 1, 365)2 -2.671391e+02 -3.306195e+02 -2.034266e+02
## t:HM                       4.696324e-02  1.255915e-02  8.618728e-02
##                              P-Value
## (Intercept)               9.718502e-15
## t                         4.379894e-13
## HM                        1.156731e-02
## rain10                    2.940639e-01
## harmonic(Month, 1, 365)1 4.503967e-16
## harmonic(Month, 1, 365)2 4.336782e-16
## t:HM                      1.159129e-02
```

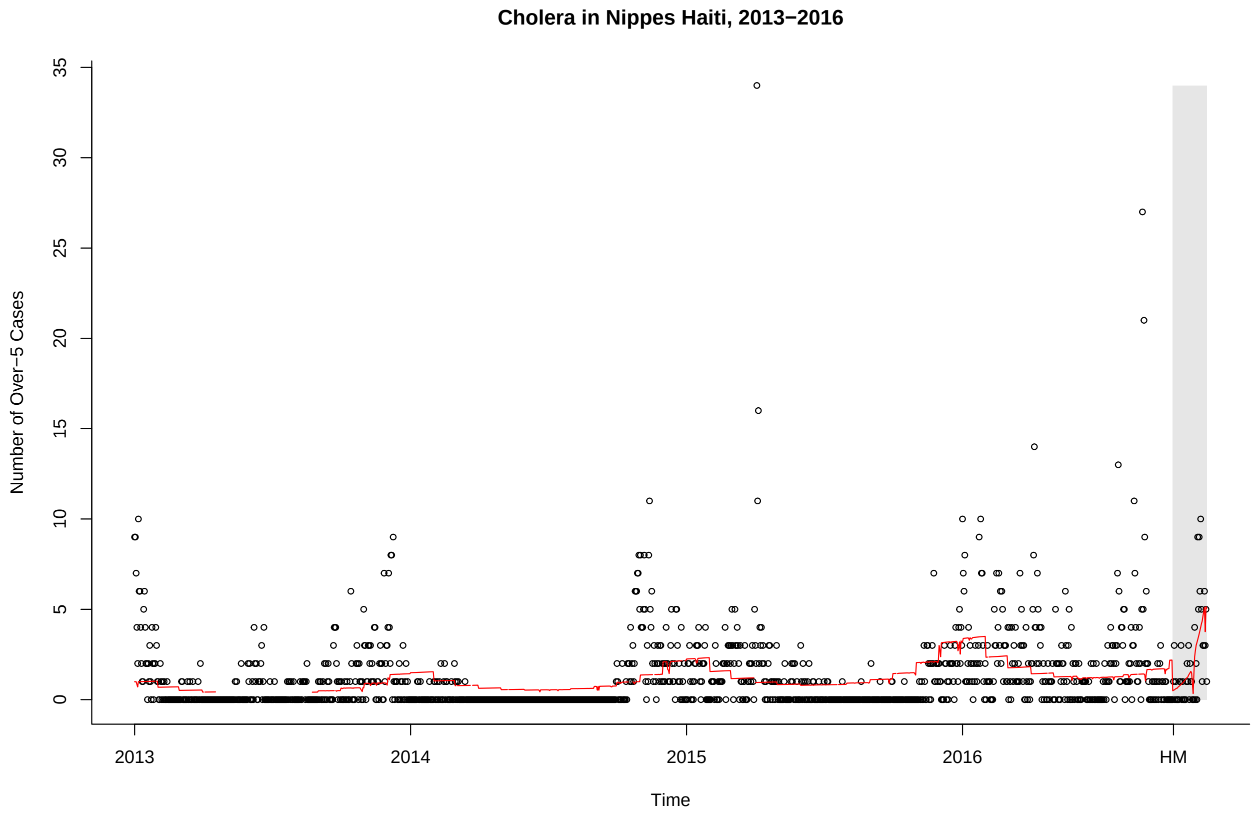

Interrupted time series regression model controlling for rainfall and seasonality, with a level only and slope-and-level change, Ouest

```
##                                Estimate       95% LCI       95% UCI
## (Intercept)                2.082680e+02  1.831039e+02  2.333840e+02
## t                          3.106365e-04  1.980379e-04  4.235507e-04
## HM                         2.302836e+01 -1.916406e+00  4.810580e+01
## rain10                     1.867991e-02 -2.844325e-02  6.074601e-02
## harmonic(Month, 1, 365)1 -2.202039e+01 -2.493075e+01 -1.910009e+01
## harmonic(Month, 1, 365)2 -2.195990e+02 -2.446322e+02 -1.945196e+02
## t:HM                      -1.700325e-02 -3.499845e-02  8.564566e-04
##                                P-Value
## (Intercept)               5.399555e-54
## t                         7.953007e-08
## HM                        7.057965e-02
## rain10                    4.104271e-01
## harmonic(Month, 1, 365)1 7.476552e-46
## harmonic(Month, 1, 365)2 1.328545e-59
## t:HM                      6.252806e-02
```

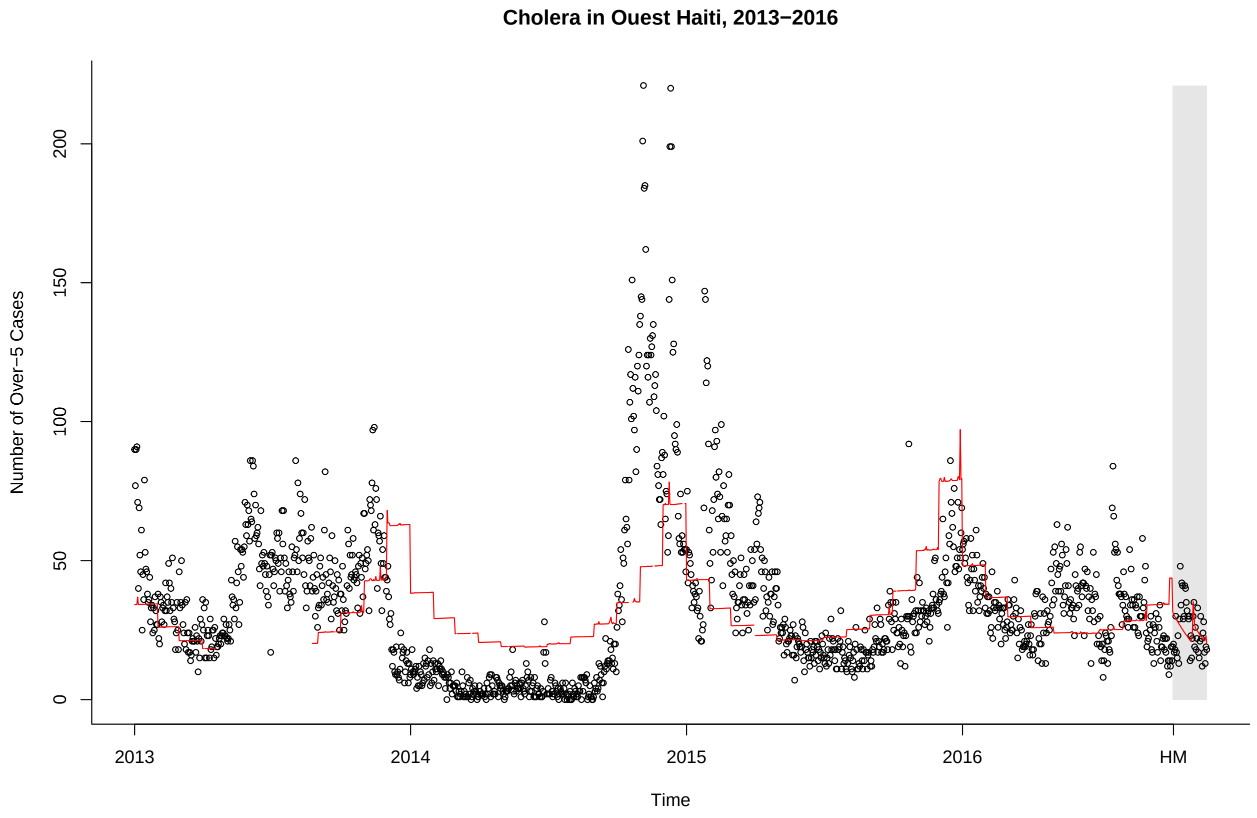

Interrupted time series regression model controlling for rainfall and seasonality, with a level only and slope-and-level change, Sud-Est

```
##                                Estimate       95% LCI       95% UCI
## (Intercept)                3.398343e+02  2.970206e+02  3.828228e+02
## t                          2.700885e-04  7.893423e-05  4.620163e-04
## HM                         3.443800e+01 -5.217480e+00  7.479174e+01
## rain10                    -7.840946e-03 -7.884614e-02  4.490228e-02
## harmonic(Month, 1, 365)1 -3.618704e+01 -4.117027e+01 -3.120873e+01
## harmonic(Month, 1, 365)2 -3.523506e+02 -3.952067e+02 -3.096731e+02
## t:HM                      -2.508261e-02 -5.407462e-02  3.302343e-03
##                               P-Value
## (Intercept)               6.625103e-50
## t                         5.784989e-03
## HM                        8.925384e-02
## rain10                    8.024722e-01
## harmonic(Month, 1, 365)1 8.365157e-43
## harmonic(Month, 1, 365)2 1.717275e-53
## t:HM                      8.437033e-02
```

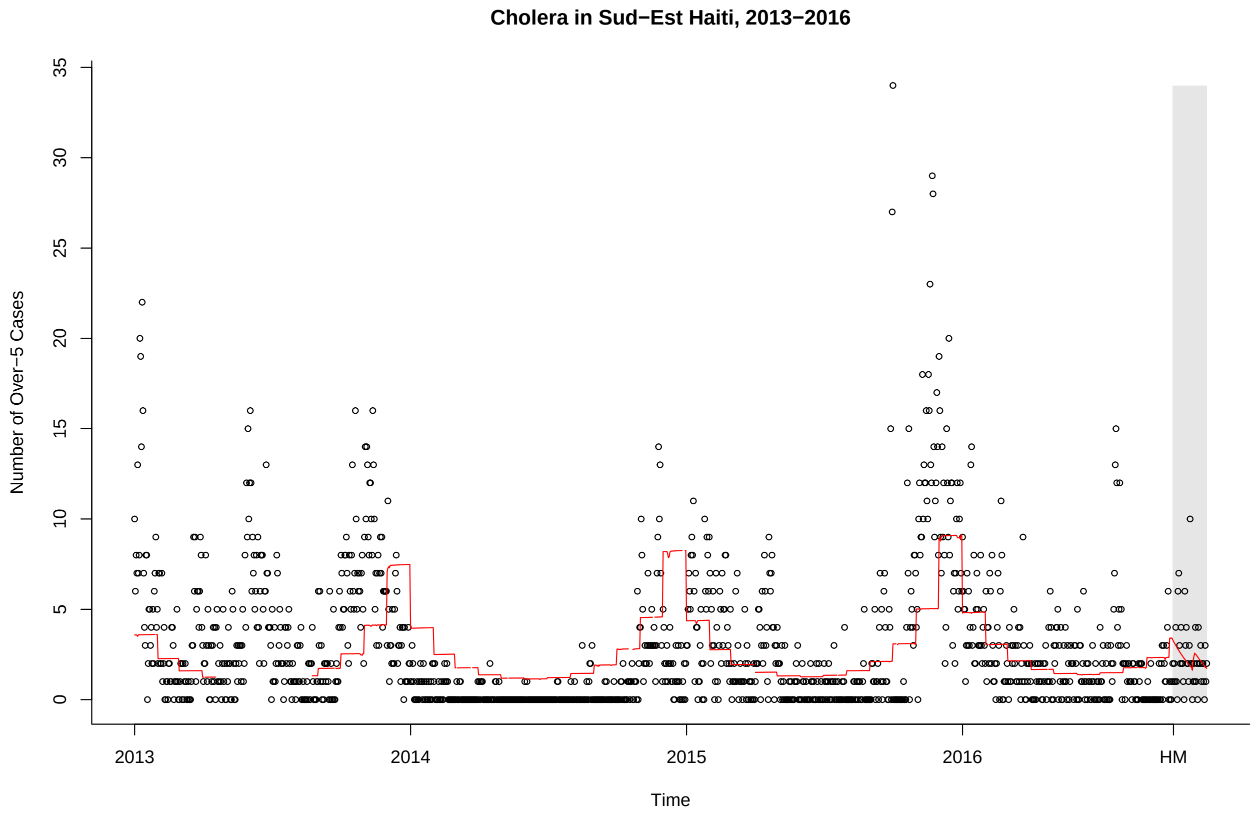
Cholera in Sud−Est Haiti, 2013−2016
Number of Over−5 Cases
0
5
10
15
20
25
30
35
2013
2014
2015
2016
HM
Time
