# Supplementary material for: Increase in Reported Cholera Cases in Haiti Following Hurricane Matthew: An Interrupted Time Series Model
Source: Am J Trop Med Hyg. 2018 Dec 26;100(2):368–73. doi: 10.4269/ajtmh.17-0964 (PMC6367609; doi:10.4269/ajtmh.17-0964)
Supplement: Supplementary file 2 [file tpmd170964.SD2.pdf]

The following are supplemental materials and will be published online only

Interrupted Time Series Regression Models for Hurricane Matthew, exploratory models

18 September 2018

Summary Statistics

National interrupted time series regression model controlling for rainfall and seasonality, with a level only and slope-and-level change, using quasi-Poisson regression with 1 Fourier Term

```
## [1] 1419
```

```
##                                        Estimate       95% LCI
## (Intercept)                        1.736513e+02  1.559676e+02
## t                                 -1.032691e-04 -1.811696e-04
## HM                                 1.962338e+01  7.297969e+00
## rain10                             1.981405e-03 -1.017957e-03
## harmonic(Month, 1, period = 365)1 -1.805041e+01 -2.009570e+01
## harmonic(Month, 1, period = 365)2 -1.848130e+02 -2.024066e+02
## t:HM                              -1.387243e-02 -2.274059e-02
##                                         95% UCI      P-Value
## (Intercept)                        1.913049e+02 1.071798e-72
## t                                 -2.535932e-05 9.480800e-03
## HM                                 3.198792e+01 1.863305e-03
## rain10                             4.718260e-03 1.751434e-01
## harmonic(Month, 1, period = 365)1 -1.599964e+01 3.473809e-60
## harmonic(Month, 1, period = 365)2 -1.671902e+02 2.498475e-81
## t:HM                              -5.042507e-03 2.151533e-03
```

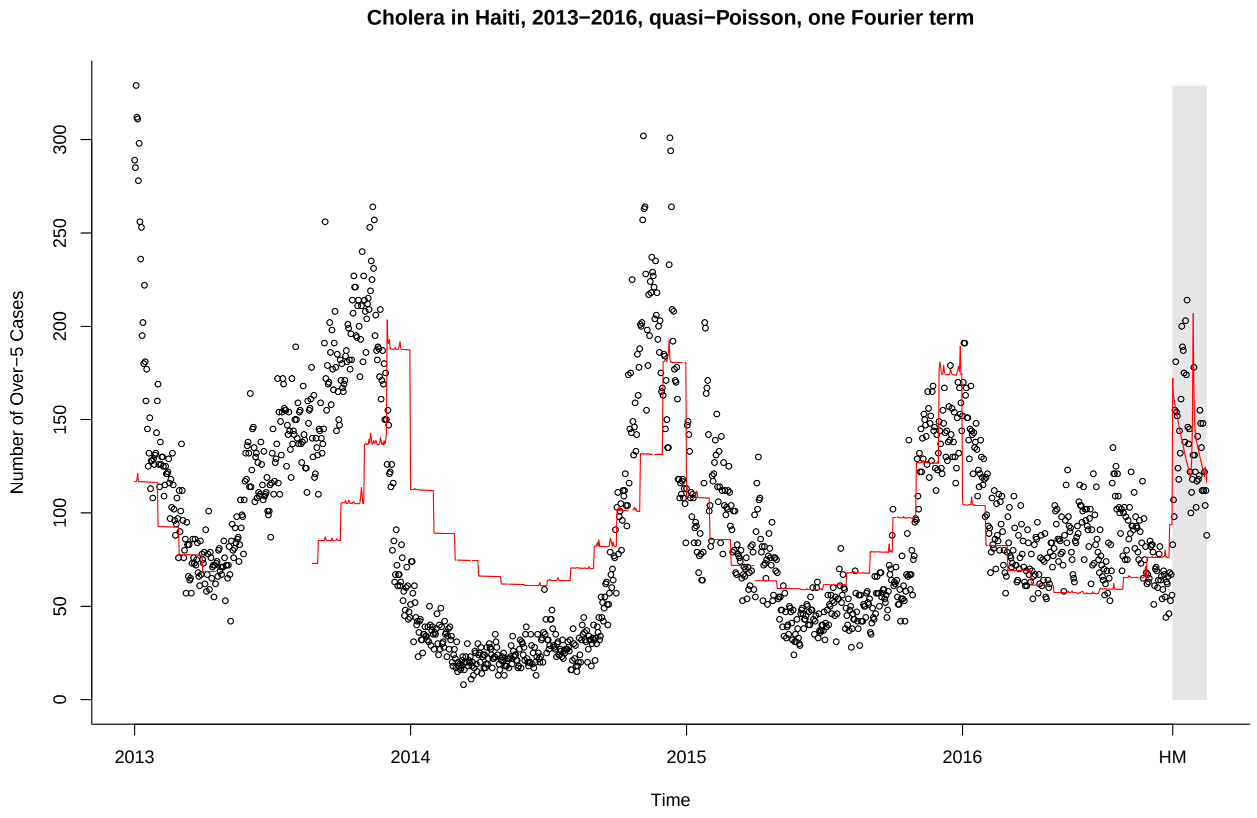

National interrupted time series regression model controlling for rainfall and seasonality, with a level only and slope-and-level change, using quasi-Poisson regression with 2 Fourier Terms

```
## [1] 1419

##                                    Estimate       95% LCI
## (Intercept)                    -8.282418e+04 -1.034199e+05
## t                              -5.205876e-05 -1.259173e-04
## HM                              1.547366e+01  3.747818e+00
## rain10                          2.110822e-03 -7.230737e-04
## harmonic(Month, 2, period = 365)1  1.080846e+04  7.720457e+03
## harmonic(Month, 2, period = 365)2 -5.401748e+03 -6.940171e+03
## harmonic(Month, 2, period = 365)3  1.100506e+05  8.276208e+04
## harmonic(Month, 2, period = 365)4 -2.723779e+04 -3.395309e+04
## t:HM                           -1.108025e-02 -1.951637e-02
##                                     95% UCI       P-Value
## (Intercept)                    -6.224511e+04 6.747637e-15
## t                               2.179134e-05 1.673384e-01
## HM                              2.723725e+01 9.881248e-03
## rain10                          4.709553e-03 1.273336e-01
## harmonic(Month, 2, period = 365)1  1.389760e+04 1.082102e-11
## harmonic(Month, 2, period = 365)2 -3.863899e+03 9.184823e-12
## harmonic(Month, 2, period = 365)3  1.373614e+05 5.971946e-15
## harmonic(Month, 2, period = 365)4 -2.052808e+04 4.006566e-15
## t:HM                           -2.680264e-03 9.951410e-03
```

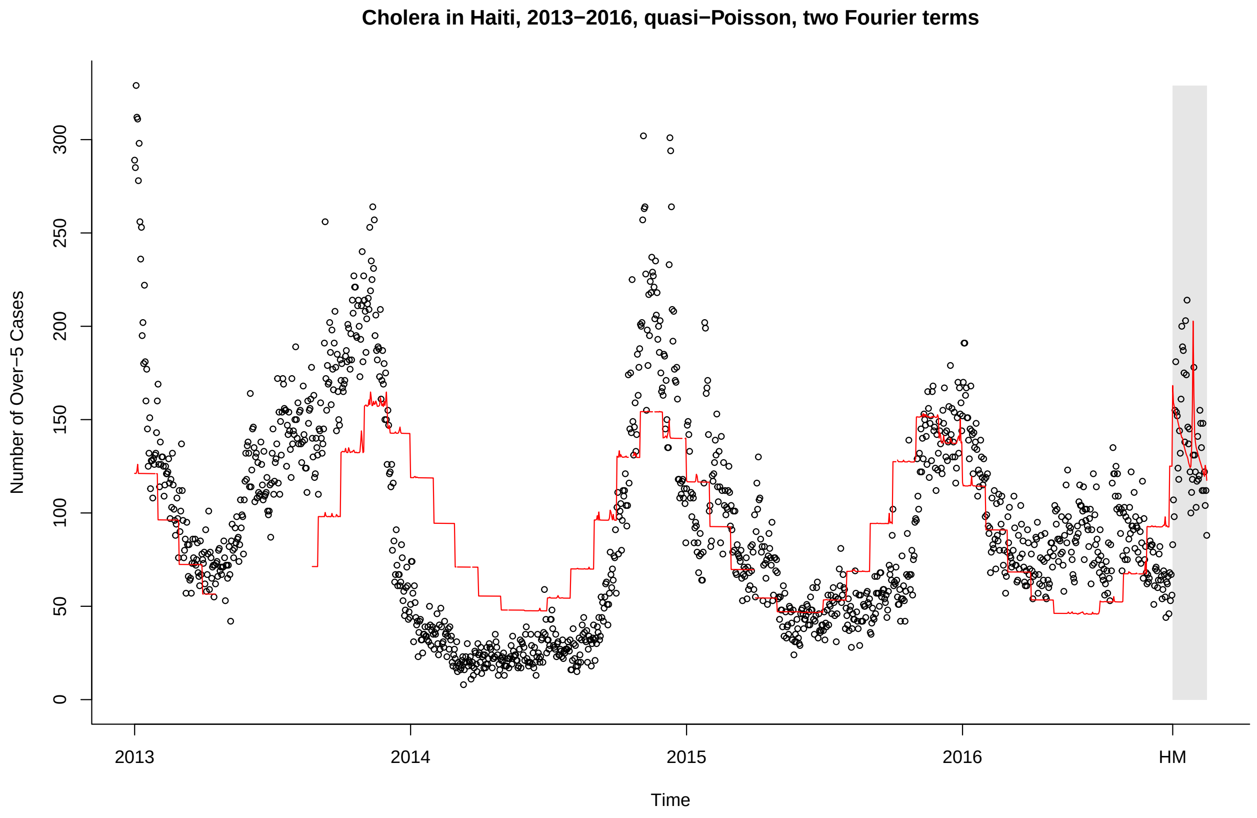


```
## Analysis of Deviance Table
##
## Model 1: CasesOver5 ~ offset(log(POP)) + t * HM + rain10 + harmonic(Month,
##     1, period = 365)
## Model 2: CasesOver5 ~ offset(log(POP)) + t * HM + rain10 + harmonic(Month,
##     2, period = 365)
##   Resid. Df Resid. Dev Df Deviance  Pr(>Chi)
## 1      1262      28810
## 2      1260      25655  2   3154.6 < 2.2e-16 ***
## ---
## Signif. codes:  0 '***' 0.001 '**' 0.01 '*' 0.05 '.' 0.1 ' ' 1
```

National interrupted time series regression model controlling for rainfall and seasonality, with a level only and slope-and-level change, using Negative Binomial regression with 1 Fourier Term

```
## [1] 1419

##                                         Estimate       95% LCI
## (Intercept)                         2.065069e+02  1.859436e+02
## t                                   1.006569e-05 -6.396075e-05
## HM                                  2.366381e+01  7.058823e+00
## rain10                              3.722410e-03 -6.011426e-04
## harmonic(Month, 1, period = 365)1 -2.134736e+01 -2.363057e+01
## harmonic(Month, 1, period = 365)2 -2.176589e+02 -2.382870e+02
## t:HM                               -1.684432e-02 -2.867512e-02
```

```
##                                            95% UCI       P-Value
## (Intercept)                           2.272009e+02  7.385902e-97
## t                                     8.395119e-05  8.059772e-01
## HM                                    4.019083e+01  3.344897e-03
## rain10                                8.552128e-03  6.408166e-02
## harmonic(Month, 1, period = 365)1 -1.907810e+01  2.867513e-81
## harmonic(Month, 1, period = 365)2 -1.971604e+02 3.987022e-108
## t:HM                                 -4.942547e-03  3.550116e-03
```

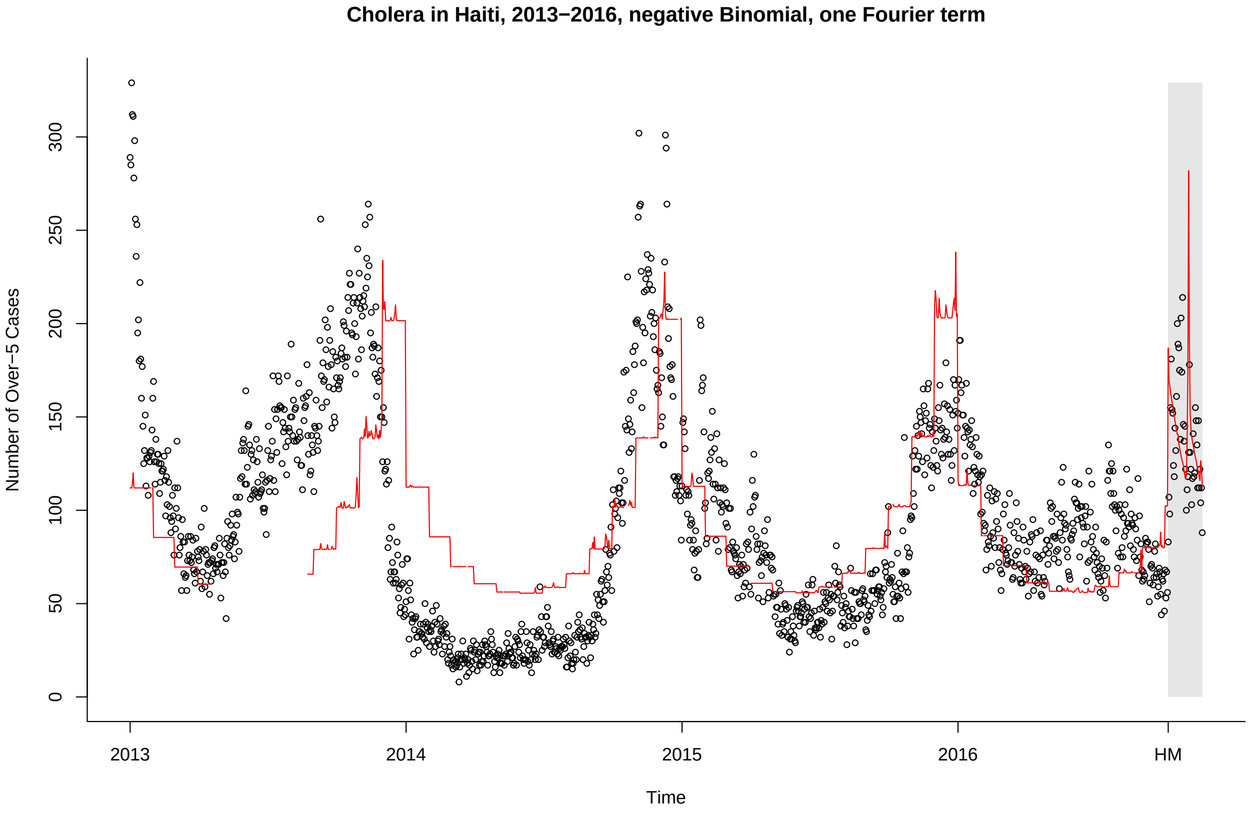

National interrupted time series regression model controlling for rainfall and seasonality, with a level only and slope-and-level change, using Negative Binomial regression with 2 Fourier Terms

```
## [1] 1419
```

```
##                                          Estimate       95% LCI
## (Intercept)                         -7.845080e+04 -1.011367e+05
## t                                     1.093969e-04  3.408071e-05
## HM                                    1.771200e+01  1.729801e+00
## rain10                                3.927867e-03 -2.656847e-04
## harmonic(Month, 2, period = 365)1  1.027324e+04  6.918496e+03
## harmonic(Month, 2, period = 365)2 -5.134857e+03 -6.805419e+03
## harmonic(Month, 2, period = 365)3  1.042396e+05  7.414062e+04
## harmonic(Month, 2, period = 365)4 -2.580026e+04 -3.320126e+04
## t:HM                                 -1.274543e-02 -2.414286e-02
##                                           95% UCI      P-Value
```

```
## (Intercept)                          -5.575563e+04 3.320944e-12
## t                                     1.846556e-04 6.339261e-03
## HM                                    3.363643e+01 2.346400e-02
## rain10                                8.596264e-03 4.320102e-02
## harmonic(Month, 2, period = 365)1  1.362757e+04 8.435314e-10
## harmonic(Month, 2, period = 365)2 -3.464093e+03 7.314225e-10
## harmonic(Month, 2, period = 365)3  1.343262e+05 3.015271e-12
## harmonic(Month, 2, period = 365)4 -1.839615e+04 2.194864e-12
## t:HM                                 -1.292549e-03 2.282468e-02
```

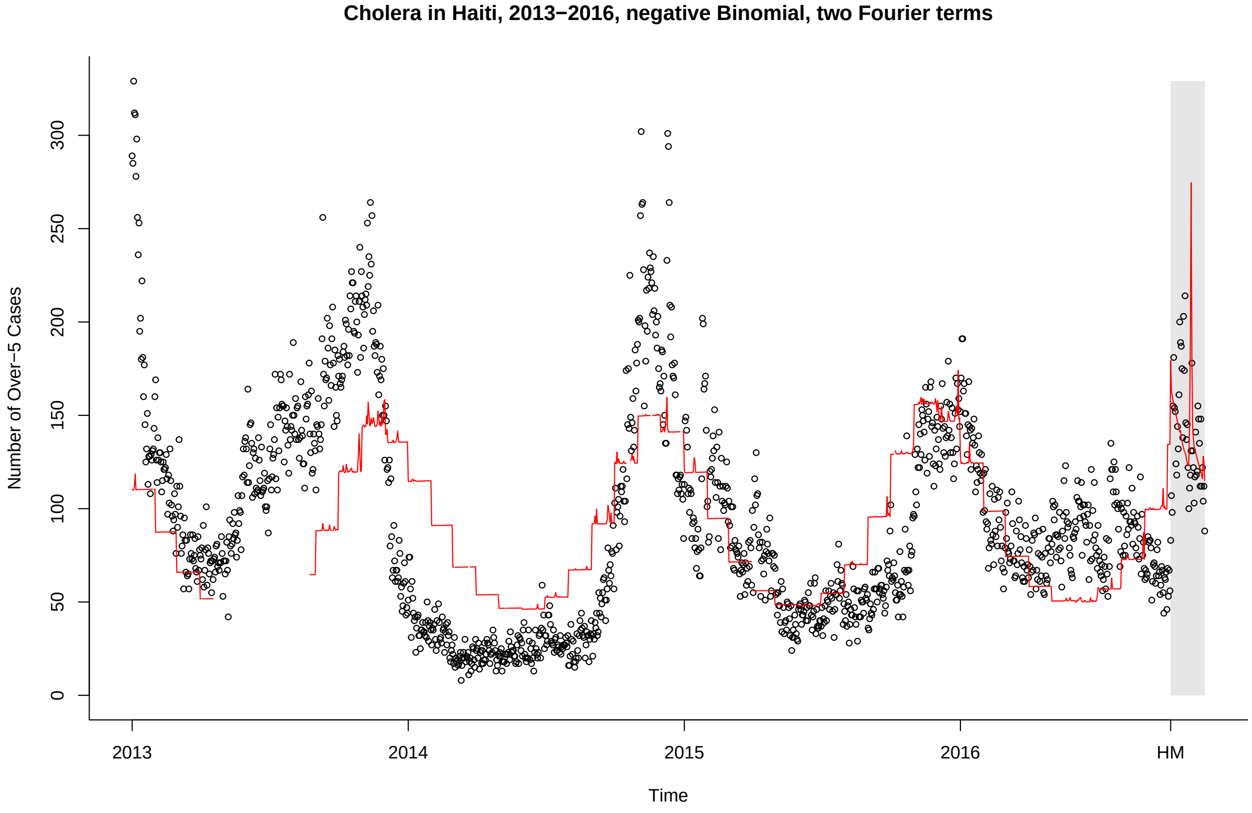


```
## Vuong Non-Nested Hypothesis Test-Statistic:
## (test-statistic is asymptotically distributed N(0,1) under the
##  null that the models are indistinguishible)
## -------------------------------------------------------------
##              Vuong z-statistic             H_A    p-value
## Raw                    -5.126751 model2 > model1 1.4739e-07
## AIC-corrected          -4.897885 model2 > model1 4.8437e-07
## BIC-corrected          -4.309013 model2 > model1 8.1992e-06
```
